# Supplementary material for: Network-based analysis reveals potential microRNA regulation of oncogenic pathways in SOX10-depleted uveal melanoma
Source: Cell Mol Life Sci. 2026 May 13;83(1):206. doi: 10.1007/s00018-026-06239-5 (PMC13168400; doi:10.1007/s00018-026-06239-5)
Supplement: Supplementary file 3 — Supplementary Material 3 (DOCX 2.25 MB) [file 18_2026_6239_MOESM3_ESM.docx]

**Supplementary Materials**

Network-based analysis reveals microRNA regulation of oncogenic pathways in SOX10-depleted uveal melanoma

Chunyan Luan^1^, Anja Wessely^1^, Zhesi Zhang^2^, Liang Zhang^2^, Adrian Weich^1,3,4^, Christopher Lischer^1^, Carola Berking^1,3,4^, Markus V. Heppt^1^, Julio Vera^1,3,4,#^, Xin Lai^1,2,5,#^

^1^Department of Dermatology, Universitätsklinikum Erlangen, CCC Erlangen-EMN, Friedrich-Alexander-Universität Erlangen-Nürnberg, 91054 Erlangen, Germany

^2^Biomedicine Unit, Faculty of Medicine and Health Technology, Tampere University, Tampere, Finland

^3^Deutsches Zentrum Immuntherapie, Erlangen, Germany

^4^Comprehensive Cancer Center Erlangen, Erlangen, Germany

^5^TAYS Cancer Centre, Tampere University Hospital, Wellbeing Services County of Pirkanmaa, Tampere, Finland

**^#^Corresponding author:**

Julio Vera ([julio.vera-gonzalez@uk-erlangen.de](mailto:julio.vera-gonzalez@uk-erlangen.de); +4991318545876), Universitätsklinikum Erlangen, Erlangen, Germany; Xin Lai ([xin.lai@tuni.fi](mailto:xin.lai@tuni.fi); +358505751673), Faculty of Medicine and Health Technology, Tampere University, Tampere, Finland.

**UM network reconstruction**

We identified the most important intracellular signaling pathways in UM through a literature survey (Table S4). We downloaded the corresponding components from the Reactome database (1) and imported them into CellDesigner (version 4.4.2). All entities were annotated directly using the Minimal Information Required In the Annotation of Models (MIRIAM) interface (2). Then, we manually expanded the network by adding a list of genes that have been previously described to interact with SOX10 (3, 4) (Table S4). We annotated proteins in the network with their UniProt IDs, genes with their Ensembl IDs, and miRNAs with their miRBase IDs. Next, we used miRNexpander to expand the manually curated network. miRNexpander is an in-house tool (https://github.com/marteber/miRNexpander) that allows us to extend human molecular interactome with databases including miRTarBase (version 6.1), miRecords (version 4.5), HTRIdb (version 1), and TRANSFAC (version 2015.1). Furthermore, we merged the UM network with our published SOX10 interaction network for the oligodendrocyte differentiation cascade (5). This allowed us to consider more SOX10-interacting proteins and their interactions. We cleaned the network using Cytoscape (version 3.8.0) by removing the duplicated nodes and self-loops. We further pruned the network using gene expression profiles. This included microarray data from 63 UM samples (GEO ACESSION ID: GSE22138) (6) and RNA sequencing data from 80 UM primary tumors (TCGA Ocular Melanomas) (7). For sequencing data, we calculated read counts and converted them to transcripts per million (TPM). Finally, we retained genes with an average expression level greater than one in either dataset. These retained genes were regarded as expressed genes in the SOX10-centered UM network.

| **Gene** | **Primer forward (5’→3’)** | **Primer reverse (5’→3’)** | **UPL probe no.** |
| --- | --- | --- | --- |
| GAPDH | AGCCACATCGCTCAGACAC | GCCCAATACGACCAAATCC | 60 |
| SOX10 | GACCAGTACCCGCACCTG | CGCTTGTCACTTTCGTTCAG | 61 |

**Table S1: Oligonucleotide primers and hydrolysis probes for qPCR.** More information about the primers used in the experiments can be found at <https://lifescience.roche.com/en_de/brands/universal-probe-library.html#assay-design-center>.


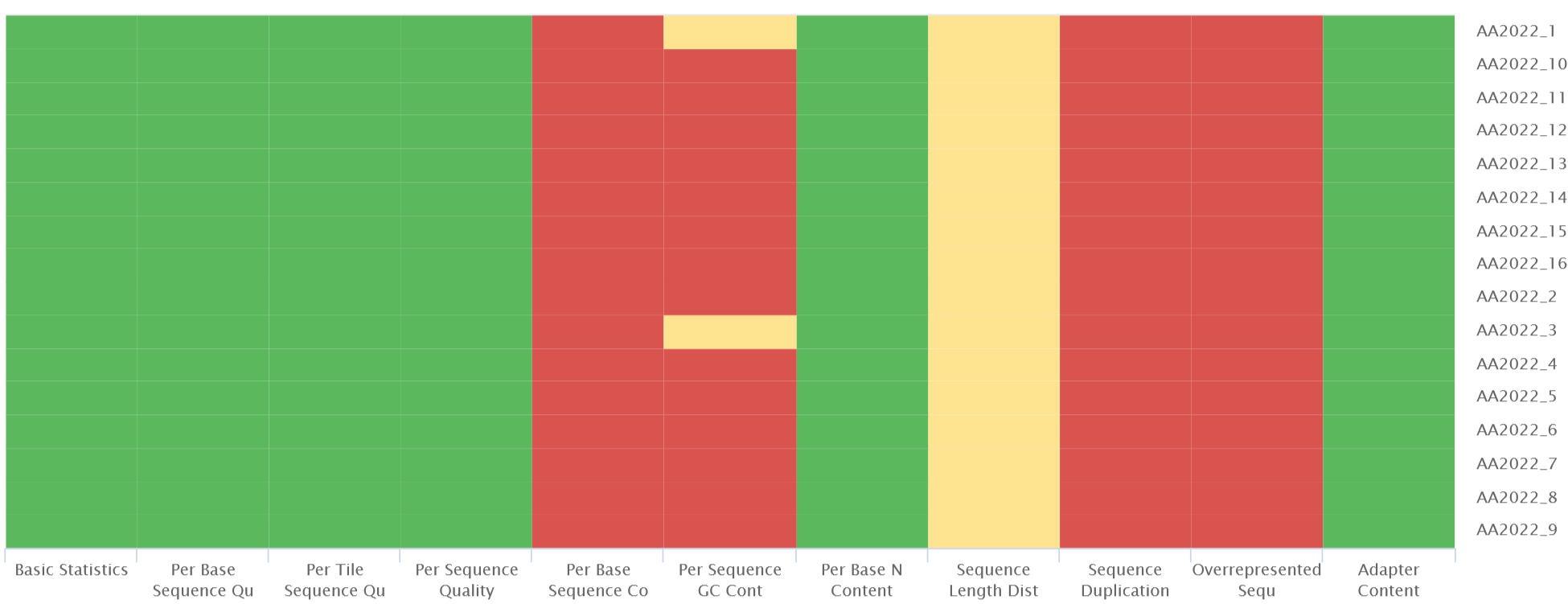


**Table S2 Qualify control of sequence reads**. The table shows quality metrics for the sequencing reads in our 16 samples. Each row is a sample (e.g. from AA2022_1 to AA2022_16) and each column represents a different quality control metric (green: completely normal; orange: slightly abnormal; red: unusual). The corresponding experimental condition for each sample can be found in Table S3. As our samples were processed with specific kits to retain small RNAs (sRNAs), we observed some abnormal and usual incidences in our samples (red and yellow grids). Our samples tend to have a distinct GC content distribution compared to total RNA sequencing results. sRNAs often have a higher GC content, which can skew the overall GC distribution. Due to the short size of sRNAs, this naturally results in a narrow sequence length distribution. In addition, certain sRNAs are highly abundant in cells (e.g., let-7 family miRNAs). This can lead to a higher proportion of overrepresented sequences. The overall diversity of sRNAs is generally lower than that of total RNA. This may lead to an increased proportion of duplicated sequences (8). The columns from left to right are - General information about the reads in each sample, such as duplicate reads, average percentage of GC content, and total number of reads per sample; Sequence quality scores per base (Phred score); Sequence quality scores per tile; Average sequence Phred score per sample; Sequence content per base; Percentage of GC content per sequence; Percentage of N bases (unknown bases) per base position; Distribution of sequence lengths; Percentage of duplicated sequences; Overrepresented sequences that appear more frequently than expected; Percentage of adapter sequences found in the reads. More detailed information for each category can be found in SM html file S1.

| **miRNA_Seq_ID** | **miRNA_Sample_ID** | **mRNA_Sample_ID** | **DE/DA_group** | **Corr_group** |
| --- | --- | --- | --- | --- |
| A2022_1 | CT_92.1_1 | S1454Nr1_CT_92.1 | CT | CT_92.1 |
| A2022_5 | CT_92.1_3 | S1454Nr3_CT_92.1 | CT | CT_92.1 |
| A2022_9 | CT_92.1_15 | S1454Nr5_CT_92.1 | CT | CT_92.1 |
| A2022_13 | CT_92.1_23 | S1454Nr7_CT_92.1 | CT | CT_92.1 |
| A2022_3 | CT_Mel_1 | S1454Nr9_CT_Mel | CT | CT_Mel |
| A2022_7 | CT_Mel_3 | S1454Nr11_CT_Mel | CT | CT_Mel |
| A2022_11 | CT_Mel_15 | S1454Nr13_CT_Mel | CT | CT_Mel |
| A2022_15 | CT_Mel_23 | S1454Nr15_CT_Mel | CT | CT_Mel |
| A2022_2 | SOX_92.1_1 | S1454Nr2_SOX_92.1 | SOX_KD | SOX_92.1 |
| A2022_6 | SOX_92.1_3 | S1454Nr4_SOX_92.1 | SOX_KD | SOX_92.1 |
| A2022_10 | SOX_92.1_15 | S1454Nr6_SOX_92.1 | SOX_KD | SOX_92.1 |
| A2022_14 | SOX_92.1_23 | S1454Nr8_SOX_92.1 | SOX_KD | SOX_92.1 |
| A2022_4 | SOX_Mel_1 | S1454Nr10_SOX_Mel | SOX_KD | SOX_Mel |
| A2022_8 | SOX_Mel_3 | S1454Nr12_SOX_Mel | SOX_KD | SOX_Mel |
| A2022_12 | SOX_Mel_15 | S1454Nr14_SOX_Mel | SOX_KD | SOX_Mel |
| A2022_16 | SOX_Mel_23 | S1454Nr16_SOX_Mel | SOX_KD | SOX_Mel |

**Table S3 Integration of miRNA and mRNA data sets**. The first column is the id used for quality control shown in Table S1. In the following two columns, each row is a sample that contains an identifier for miRNA and mRNA samples. The identifier includes experimental condition (CT: control; SOX10 knockdown: SOX_KD) and cell lines (92.1 and Mel). The last two columns show the grouped samples, and the information is used for differential expression (DE), differential activity (DA), and correlation (Corr) analysis.

| Name/Pathway | Description or ID | Reference |
| --- | --- | --- |
| SOX10-interacting genes | ETS1, E2F1, TYRP1, NES, EDNRB, PAX3, RET, EGR2, POU3F1, POU3F2, SP1, MED1, MITF, DCT, TYR, KIT, IRF4, MEF2C, ERBB3, ITPR2, CEBP, CREB3L2, BHLHB2 | (3, 4) |
| CD47 | R-HSA-391160 | (14, 15) |
| DNA double-strand break repair | R-HSA-5693532 | (9–11) |
| FasL | R-HSA-75157 | (14, 15) |
| Hypoxia response | R-HSA-1234174 | (7, 9) |
| IL-10 | R-HSA-6783783 | (14, 15) |
| IFN-gamma | R-HSA-877300 | (14, 15) |
| MIF | R-HSA-8950732 | (14, 15) |
| PD-1 | R-HSA-389948 | (14, 15) |
| P13K-Akt-mTOR | R-HSA-109704, R-HSA-165159, R-HSA-6807070 | (11, 13) |
| PLC-PKC | R-HSA-418217 | (7, 9, 13) |
| Ras-Raf-MEK-ERK-MAPK | R-HSA-5673001, R-HSA-416476 | (7, 9, 10, 12, 13) |
| SOX10 | R-HSA-9619665 | (4, 16) |
| Trio-Rho-Rac | R-HSA-9012999 | (13) |
| TGF-beta | R-HSA-170834 | (14, 15) |
| YAP-Hippo | R-HSA-2028269 | (12) |

**Table S4: Intracellular pathways of UM and SOX10 interacting genes.** The first row is a list of known SOX10 interacting genes. The table contains three columns that are pathway names and ids from Reactome and corresponding references.


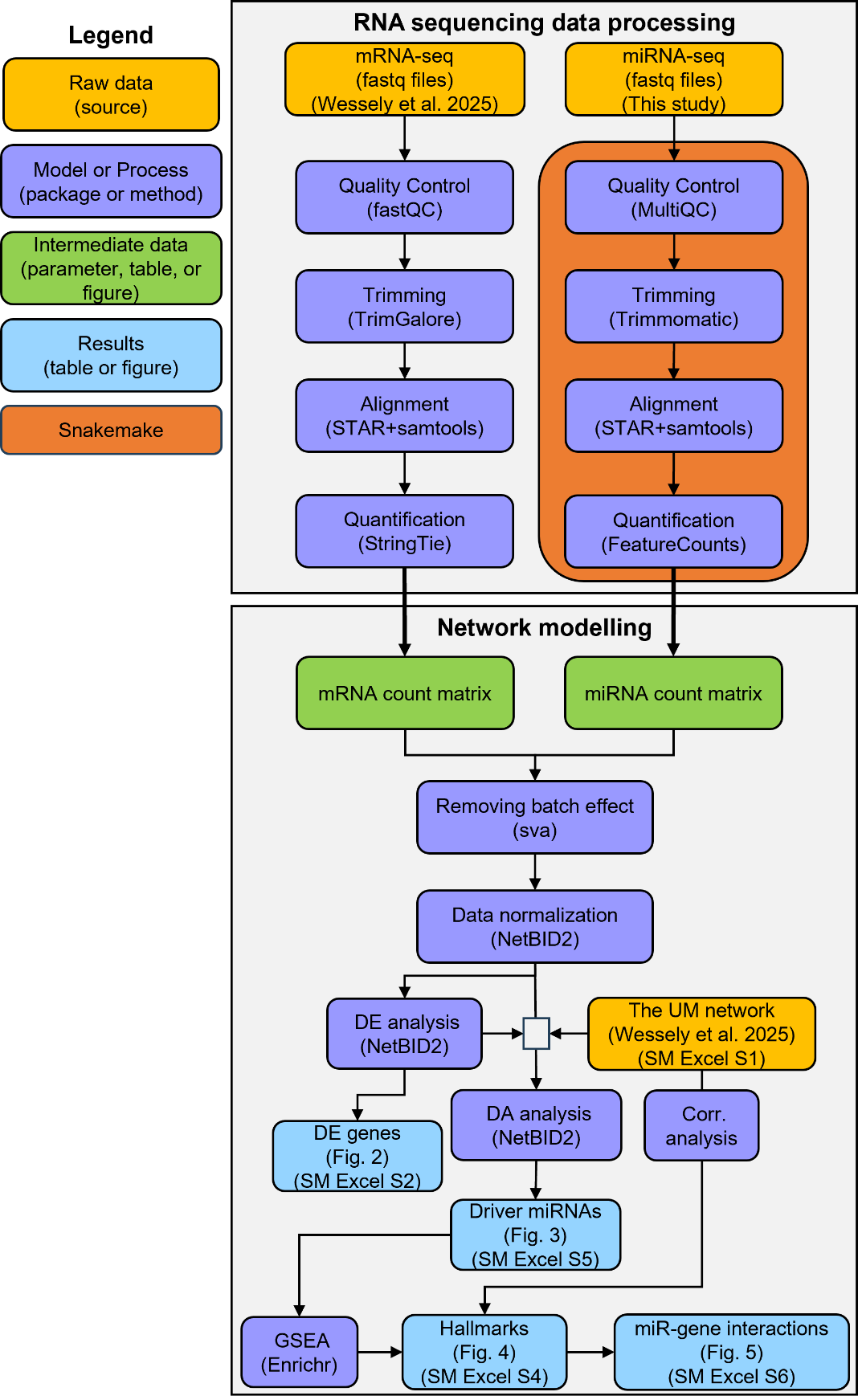


**Figure S1 The detailed computational workflow**. The approach contains several modules (grey blocks). In each module, we show the data (yellow rectangles), method and model used for analysis (purple rectangles), intermediate data (green rectangles), and results (blue rectangles). The shaded area highlighted in orange is implemented using Snakemake v9.8. The communication between different parts of a module is connected using thin arrowed lines. Cross communication between modules is connected using thin and thick arrowed lines. The texts in the parentheses are the corresponding source of the data, the packages and methods used by us, tables (Tab.), or figures (Fig.) presented in the article. DE: differentially expressed. GSEA: gene set enrichment analysis. UM: uveal melanoma. The mRNA data and UM network used were from Wessely et al.2025 (17).


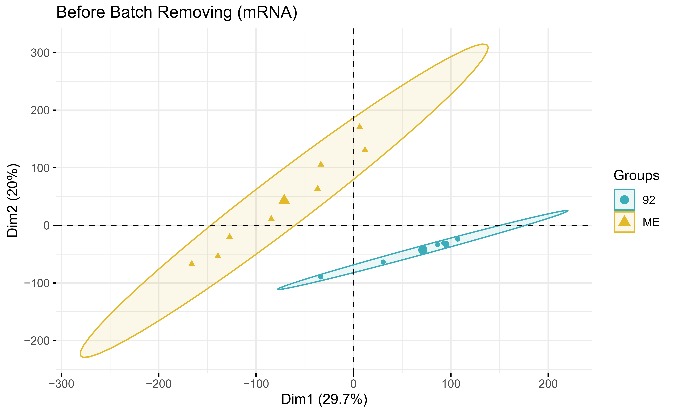

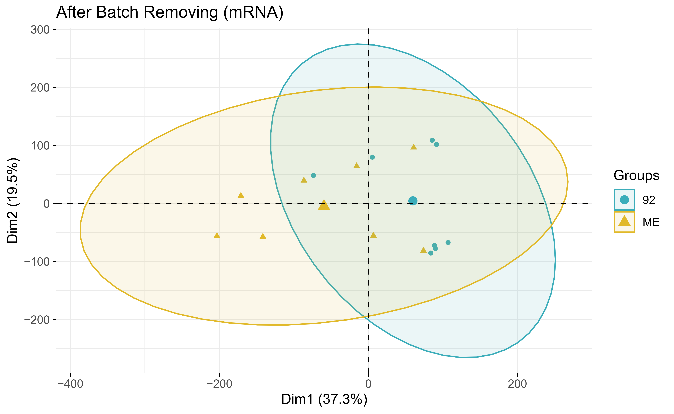


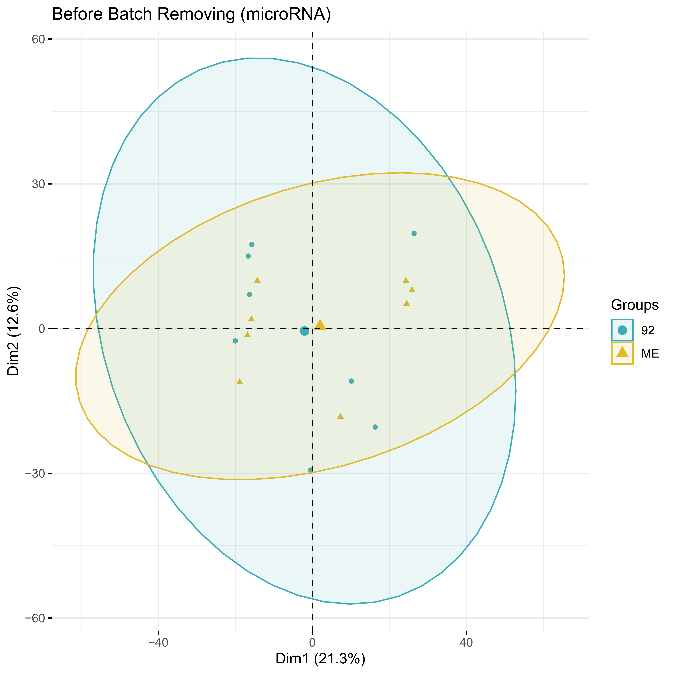

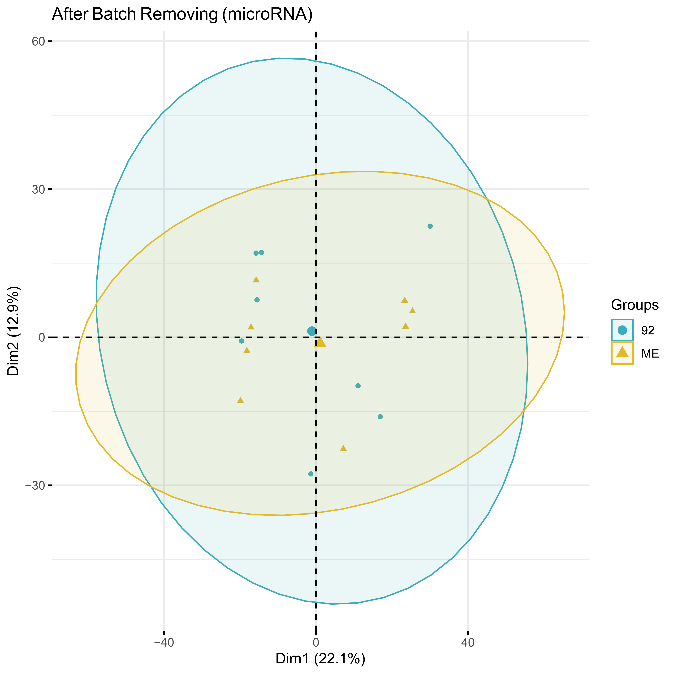


**Figure S2 Batch effect analysis**. The figures show the results of PCA before (**left**) and after (**right**) removing batch effects in mRNA (**top**) and miRNA (**bottom**) sequencing data. The small blue circles and orange triangles represent individual samples from two UM cell lines (92.1 and Mel270), respectively. The big circles and triangles are the centers of the two cell lines. The ellipses represent the confidence areas of the cell lines.


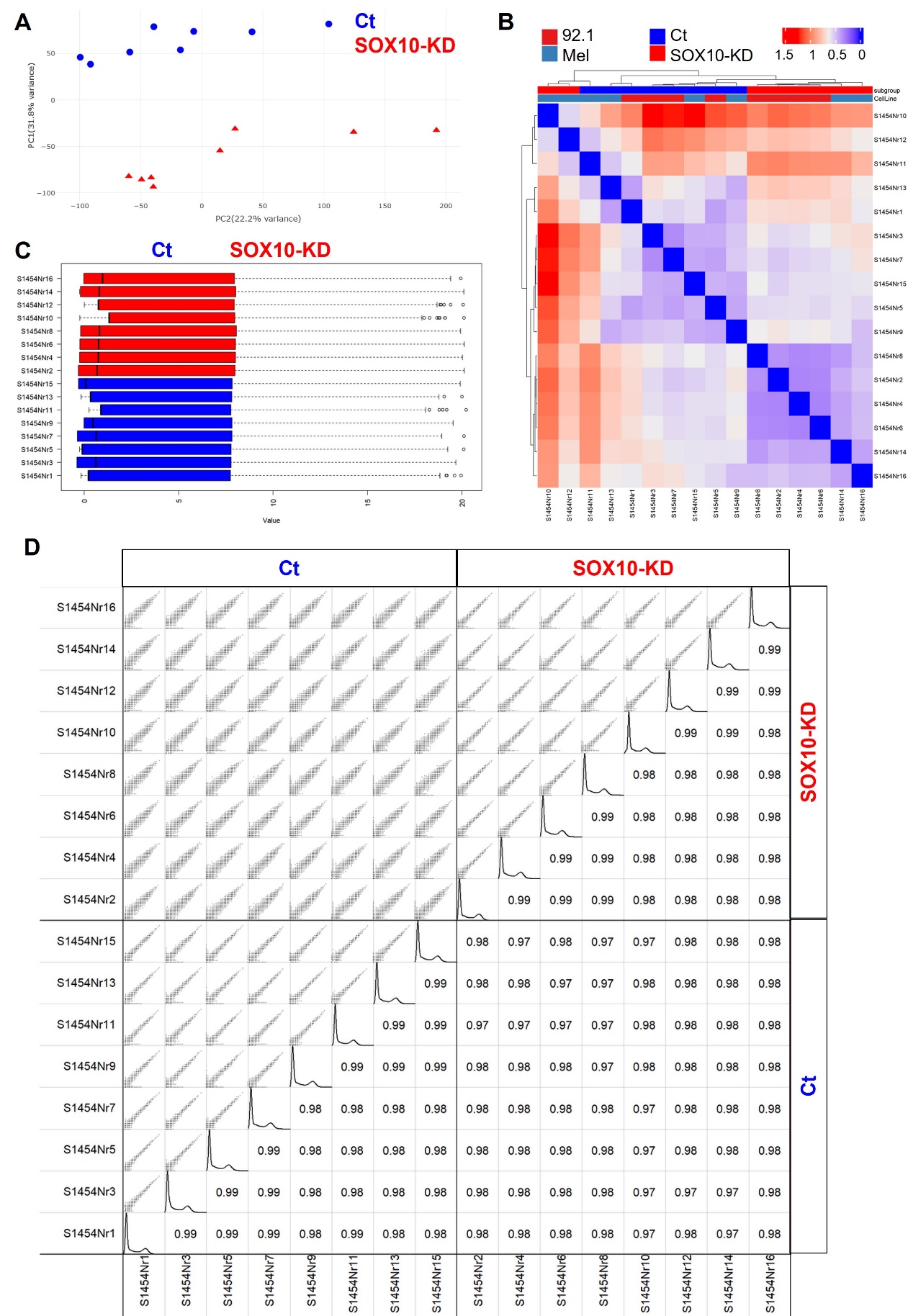


**Figure S3 Overview of the merged miRNA and mRNA data**. (**A**) The PCA plot after merging the mRNA and miRNA data. (**B**) The heat map shows the clustering of samples based on the distance of their gene expression profiles. (**C**) The expression value (i.e. log2(counts)) distribution for each sample. (**D**) The correlation analysis for all samples shown by scattering plots (the upper part of the diagonal line), density plot (the diagonal line), and correlation coefficients (the lower part of the diagonal line.


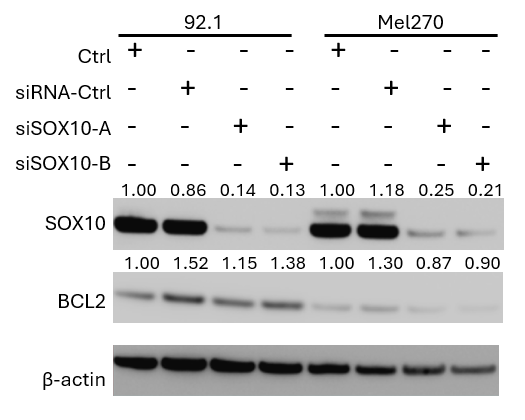


**Figure S4 Downregulation of BCL2 in SOX10-KD UM**. The Western blot shows the downregulation of SOX10 by siRNA and its impact on BCL2 expression levels in UM. The data were first quantified using ImageJ and normalized to β-actin, and then to Ctrl. The knockdown of SOX10 was achieved using siSOX10-A or siSOX10-B. We measured the protein expression of SOX10 and BCL2 in the 92.1 and Mel270 cell lines 48 hours after they were transfected with a control (Ctrl), which involved treating the cells with the transfection reagent Lipofectamine RNAiMAX only, control siRNA (siRNA-Ctrl), siSOX10-A, or siSOX10-B. β-actin served as the loading control.


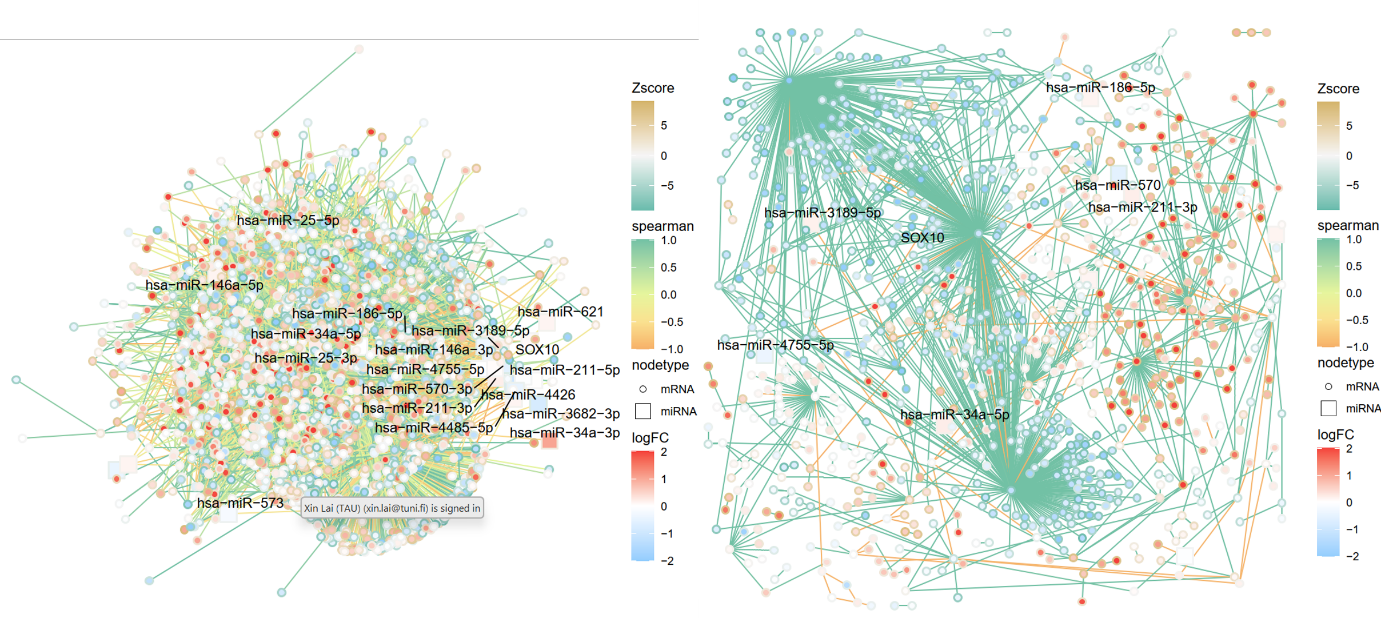


**Figure S5 The UM network**. The UM network shows regulatory interactions among miRNAs and their targets. miRNAs and PCGs are shown in squares and circles, respectively. Node colors are log2 fold-change of gene expression levels and node borders represent genes’ regulatory activity change in the z-score scale. Edge colors represent the Spearman correlation coefficients between interacting genes. For visualization, we filter out edges with absolute correlation values smaller than 0.9 and highlight only significant driver miRNAs.

**Reference**

1. Milacic,M., Beavers,D., Conley,P., Gong,C., Gillespie,M., Griss,J., Haw,R., Jassal,B., Matthews,L., May,B., *et al.* (2024) The Reactome Pathway Knowledgebase 2024. *Nucleic Acids Res*, **52**, D672–D678.

2. Le Novère,N., Finney,A., Hucka,M., Bhalla,U.S., Campagne,F., Collado-Vides,J., Crampin,E.J., Halstead,M., Klipp,E., Mendes,P., *et al.* (2005) Minimum information requested in the annotation of biochemical models (MIRIAM). *Nat Biotechnol*, **23**, 1509–1515.

3. Harris,M.L., Baxter,L.L., Loftus,S.K. and Pavan,W.J. (2010) Sox proteins in melanocyte development and melanoma. *Pigment Cell Melanoma Res*, **23**, 496–513.

4. Fufa,T.D., Harris,M.L., Watkins-Chow,D.E., Levy,D., Gorkin,D.U., Gildea,D.E., Song,L., Safi,A., Crawford,G.E., Sviderskaya,E.V., *et al.* (2015) Genomic analysis reveals distinct mechanisms and functional classes of SOX10-regulated genes in melanocytes. *Hum Mol Genet*, **24**, 5433–5450.

5. Cantone,M., Küspert,M., Reiprich,S., Lai,X., Eberhardt,M., Göttle,P., Beyer,F., Azim,K., Küry,P., Wegner,M., *et al.* (2019) A gene regulatory architecture that controls region-independent dynamics of oligodendrocyte differentiation. *Glia*, **67**, 825–843.

6. Laurent,C., Valet,F., Planque,N., Silveri,L., Maacha,S., Anezo,O., Hupe,P., Plancher,C., Reyes,C., Albaud,B., *et al.* (2011) High PTP4A3 phosphatase expression correlates with metastatic risk in uveal melanoma patients. *Cancer Res*, **71**, 666–674.

7. Robertson,A.G., Shih,J., Yau,C., Gibb,E.A., Oba,J., Mungall,K.L., Hess,J.M., Uzunangelov,V., Walter,V., Danilova,L., *et al.* (2017) Integrative Analysis Identifies Four Molecular and Clinical Subsets in Uveal Melanoma. *Cancer Cell*, **32**, 204-220.e15.

8. Fuchs,R.T., Sun,Z., Zhuang,F. and Robb,G.B. (2015) Bias in ligation-based small RNA sequencing library construction is determined by adaptor and RNA structure. *PLoS One*, **10**, e0126049.

9. Amaro,A., Gangemi,R., Piaggio,F., Angelini,G., Barisione,G., Ferrini,S. and Pfeffer,U. (2017) The biology of uveal melanoma. *Cancer Metastasis Rev*, **36**, 109–140.

10. Johansson,P.A., Brooks,K., Newell,F., Palmer,J.M., Wilmott,J.S., Pritchard,A.L., Broit,N., Wood,S., Carlino,M.S., Leonard,C., *et al.* (2020) Whole genome landscapes of uveal melanoma show an ultraviolet radiation signature in iris tumours. *Nat Commun*, **11**, 2408.

11. Singh,M., Durairaj,P. and Yeung,J. (2018) Uveal Melanoma: A Review of the Literature. *Oncol Ther*, **6**, 87–104.

12. Piaggio,F., Tozzo,V., Bernardi,C., Croce,M., Puzone,R., Viaggi,S., Patrone,S., Barla,A., Coviello,D., Jager,M.J., *et al.* (2019) Secondary Somatic Mutations in G-Protein-Related Pathways and Mutation Signatures in Uveal Melanoma. *Cancers (Basel)*, **11**, 1688.

13. Wessely,A., Steeb,T., Erdmann,M., Heinzerling,L., Vera,J., Schlaak,M., Berking,C. and Heppt,M.V. (2020) The Role of Immune Checkpoint Blockade in Uveal Melanoma. *Int J Mol Sci*, **21**, 879.

14. Basile,M.S., Mazzon,E., Fagone,P., Longo,A., Russo,A., Fallico,M., Bonfiglio,V., Nicoletti,F., Avitabile,T. and Reibaldi,M. (2019) Immunobiology of Uveal Melanoma: State of the Art and Therapeutic Targets. *Front Oncol*, **9**, 1145.

15. E,R., G,S., Ig,Z., Ba,M., Mm,P., Mg,S., V,F., G,P., A,C., G,R., *et al.* (2019) Immunological Backbone of Uveal Melanoma: Is There a Rationale for Immunotherapy? *Cancers*, **11**.

16. Harris,M.L., Baxter,L.L., Loftus,S.K. and Pavan,W.J. (2010) Sox proteins in melanocyte development and melanoma. *Pigment Cell Melanoma Res*, **23**, 496–513.

17. Wessely,A., Lischer,C., Weich,A., Kammerbauer,C., Güse,E., Koch,E.A.T., Lai,X., Dörrie,J., Erdmann,M., Voskens,C., *et al.* (2025) A computational SOX10 network-based selection strategy to identify new drug targets in uveal melanoma. 10.1101/2025.10.14.679939.
